# Tick infestation in birds and prevalence of pathogens in ticks collected from different places in Germany

**DOI:** 10.1007/s00436-016-5022-5

**Published:** 2016-04-06

**Authors:** Christine Klaus, Jörn Gethmann, Bernd Hoffmann, Ute Ziegler, Martin Heller, Martin Beer

**Affiliations:** Friedrich-Loeffler-Institute, Institute of Bacterial Infections and Zoonoses, Naumburger Str. 96a, D-07743 Jena, Germany; Friedrich-Loeffler-Institute, Institute of Epidemiology, Greifswald, Insel Riems Germany; Friedrich-Loeffler-Institute, Institute of Diagnostic Virology, Greifswald, Insel Riems Germany; Friedrich-Loeffler-Institute, Institute of Novel and Emerging Diseases, Greifswald, Insel Riems Germany; Friedrich-Loeffler-Institute, Institute of Molecular Pathogenesis, Jena, Germany

**Keywords:** Tick-borne encephalitis virus, Ticks, Birds, Bird migration, *Ixodes* spp., *Borrelia* spp.

## Abstract

The importance of ticks and tick-borne pathogens for human and animal health has been increasing over the past decades. For their transportation and dissemination, birds may play a more important role than wingless hosts. In this study, tick infestation of birds in Germany was examined. Eight hundred ninety-two captured birds were infested with ticks and belonged to 48 different species, of which blackbirds (*Turdus merula*) and song thrushes (*Turdus philomelos*) were most strongly infested. Ground feeders were more strongly infested than non-ground feeders, sedentary birds more strongly than migratory birds, and short-distance migratory birds more strongly than long-distance migratory birds. Mean tick infestation per bird ranged between 2 (long-distance migratory bird) and 4.7 (sedentary bird), in some single cases up to 55 ticks per bird were found. With the exception of three nymphs of *Haemaphysalis* spp., all ticks belonged to *Ixodes* spp., the most frequently detected tick species was *Ixodes ricinus*. Birds were mostly infested by nymphs (65.1 %), followed by larvae (32.96 %). Additionally, ticks collected from birds were examined for several pathogens: Tick-borne encephalitis virus (TBEV) and Sindbisvirus with real-time RT-PCR, Flaviviruses, Simbuviruses and Lyssaviruses with broad-range standard RT-PCR-assays, and *Borrelia* spp. with a Pan-*Borrelia* real-time PCR. Interestingly, no viral pathogens could be detected, but *Borrelia* spp. positive ticks were collected from 76 birds. *Borrelia* (*B*.) *garinii*, *B. valaisiaina*, *B. burgdorferi s.s*. and *B. afzelii* were determined. The screening of ticks and birds for viral pathogens with broad range PCR-assays was tested and the use as an “early warning system” is discussed.

## Introduction

Arthropod-borne and especially tick-borne diseases have become a growing public health concern over the past decades (World Health Organization 2004, 2014) and cause severe health problems in humans and animals (Estrada-Peña 2009). In order to maintain tick-borne diseases in a population, it is essential that the pathogen, a susceptible vertebrate host, and ticks with competence for the pathogen are available. Many different biotic and abiotic factors can influence these three components and all components must be available in sufficient numbers at the same time and the same place as described e.g., by Pfeffer and Dobler (2010). In nature, for new and emerging diseases, the chance for such a scenario is most likely very low. Nevertheless, outbreaks of vector-borne animal diseases like bluetongue disease or Schmallenberg virus infection in Europe (Wilson and Mellor 2009; Hoffmann et al. 2012) as well as Usutu virus infection in Germany (Becker et al. 2012) have shown that this scenario is reality, and in some cases cannot only cause severe medical problems but also financial losses in agriculture.

The spread of ticks and tick-borne pathogens can be promoted by global trade, animal migration (Pfeffer and Dobler 2010), and climate change (Estrada-Peña 2009). In this context, migratory birds play a more important role than less mobile wingless hosts (Hasle 2013).

In Germany, only a small number of studies have been carried out to investigate ticks from birds for tick-borne pathogens, and so far no data are available about tick infestation of birds. Franke et al. (2010a, b) checked 221 and 191 ticks, respectively, from birds for bacterial pathogens and detected *Borrelia* spp., *Anaplasma phagocytophilum*, *Rickettsia* spp., *Babesia* spp. and *Francisella tularensis*.

Initially, our aim was to determine the number of tick-carrying birds in Germany. It was hypothesized that tick infestation depends on species and age and the biology of the individual bird species, migratory bird species were compared to sedentary bird species and ground feeder species were compared to non-ground feeder species.

Except for TBEV, knowledge about tick-borne viral pathogens in birds in Germany is limited. It is expected that viral infection is more likely in adult ticks or nymphs than in larvae. In addition to tick infestation of birds, adult ticks and nymphs were checked for viral pathogens. Ticks were investigated by various real-time PCR systems for detection of whole virus genera or phylogenetic groups as a screening test that is less time-consuming and costly than species specific PCR systems, and with some specific real-time PCR tests for viral pathogens that are common in Germany, such as TBEV and Sindbisvirus.

Finally, adult ticks and nymphs were tested for *Borrelia* spp., the most common tick-borne pathogen in Germany.

## Materials and methods

From July 2008 to December 2010, data on captured birds and collected ticks were provided by 25 persons or groups of persons in six federal states in Germany. Nine of them did not send in a complete dataset (ticks and total number of birds caught); hence, we had to exclude them from the risk factor analysis for tick infestation. Nevertheless, these ticks were included in the following parts of our study about the screening for tick species and tick-borne pathogens. The remaining persons caught birds in five different federal states (Table 1). Birds were caught in mist nets or in Helgoland traps and were identified by species and ring number. If possible, sex and age were determined. Bird species were allocated to migratory birds and differentiated as long-distance migratory birds which overwinter in Sub-Saharan Africa, and short-distance migratory birds which overwinter in Western Europe and Northern Africa, and sedentary birds that stay in the same region year-round. Ticks were removed by special tweezers, collected in plastic vials, frozen at −20 °C, transported to the lab, and differentiated according to Estrada-Peña et al. (2004), Pfister (2006) or Eckert et al. (2008), and finally stored at −80 °C for further investigations.

**Table 1:** Places of tick collection from birds

Country	District	Place	Number sampled	Number ringed
Baden-Württemberg	Konstanz	Radolfzell	82	7002
Baden-Württemberg	Stuttgart	Stuttgart	29	467
Brandenburg	Potsdam	Potsdam	7	321
Brandenburg	Havelland	Rathenow	22	882
Saxony-Anhalt	Anhalt-Bitterfeld	Steckby	55	492
Saxony-Anhalt	Magdeburg	Magdeburg	33	1828
Saxony	Erzgebirgskreis	Annaberg-Buchholz	73	1502
Saxony	Dresden	Dresden	65	2350
Saxony	Leipzig	Grimma	1	419
Saxony	Leipzig	Leipzig	48	695
Saxony	Leipzig	Leipzig	7	835
Saxony	Bautzen	Lohsa	8	294
Saxony	Bautzen	Pulsnitz	12	857
Thuringia	Unstrut-Hainich	Mülverstedt	152	2406
Thuringia	Sonneberg	Steinheid	14	2039
Thuringia	Eichsfeld	Wachstedt	117	1560
Brandenburg	Havelland	Buckow-Nennhausen	14	^a^
Brandenburg	Uckermark	Lychen	5	^a^
Hesse	Frankfurt	Frankfurt	37	^a^
Hesse	Frankfurt	Frankfurt	50	^a^
Hesse	Hochtaunuskreis	Oberursel	5	^a^
Saxony-Anhalt	Magdeburg	Magdeburg	2	^a^
Saxony-Anhalt	Halle	Halle	9	^a^
Saxony	Leipzig	Markranstädt	8	^a^
Saxony	Dresden	Dresden	37	^a^
Summary			892	
Summary data for analysis 1			725	23,949

^a^Excluded from analysis 1: tick-infested birds compared to all ringed birds

Data of birds and ticks from birds were statistically analysed with the R statistical software 3.1.1., using Fisher exact test (fisher.test {stats}) and MS Excel 2010.

Classification into ground feeders and non-ground feeders is not always clear because most bird species have main places where they search for food and visit both, ground and non-ground places. Additionally, the places where birds mainly find food depend on the season. In this study, classification was done regarding the tick season for sedentary birds and the breeding season for migratory birds. In addition to the statistical analysis of the registered data sets from ticks and birds, in adult ticks and nymphs arthropod- and bird-associated viral pathogens as well as *Borrelia* spp. as the most common tick-borne bacterial pathogen in Germany were investigated. DNA and RNA were extracted as follows: Ticks were ground up in a mixer mill with three stainless steel beads (Retsch GmbH, Haan, Germany) and 400 μl medium (MEM Earle, Biochrom AG, Berlin, Germany). For DNA and RNA extraction, pools of five tick suspensions were generated with 100 μl of each tick suspension. The remaining sample was stored for further investigation of single ticks in case of pathogen detection in some of the pools. The pools were used for DNA and RNA extraction according to the manufacturer’s instructions using the NucleoSpin® 96 Virus kit (Macherey-Nagel, Düren, Germany). Detection of viral pathogens was performed by using the TBEV-real-time RT-PCR according to Schwaiger and Cassinotti (2003, modified by Klaus et al. 2010), a Flavivirus-RT-PCR (Johnson et al. 2010), a Simbuvirus-RT-qPCR (Fischer et al. 2013), a Lyssavirus-RT-qPCR (Fischer et al. 2014), and a Sindbisvirus-RT-qPCR (Jöst et al. 2010). Genome detection of *Borrelia* spp. was performed by Pan-Borrelia real-time PCR according to Strube et al. (2010).

## Results

### Birds

**Fig. 1:**
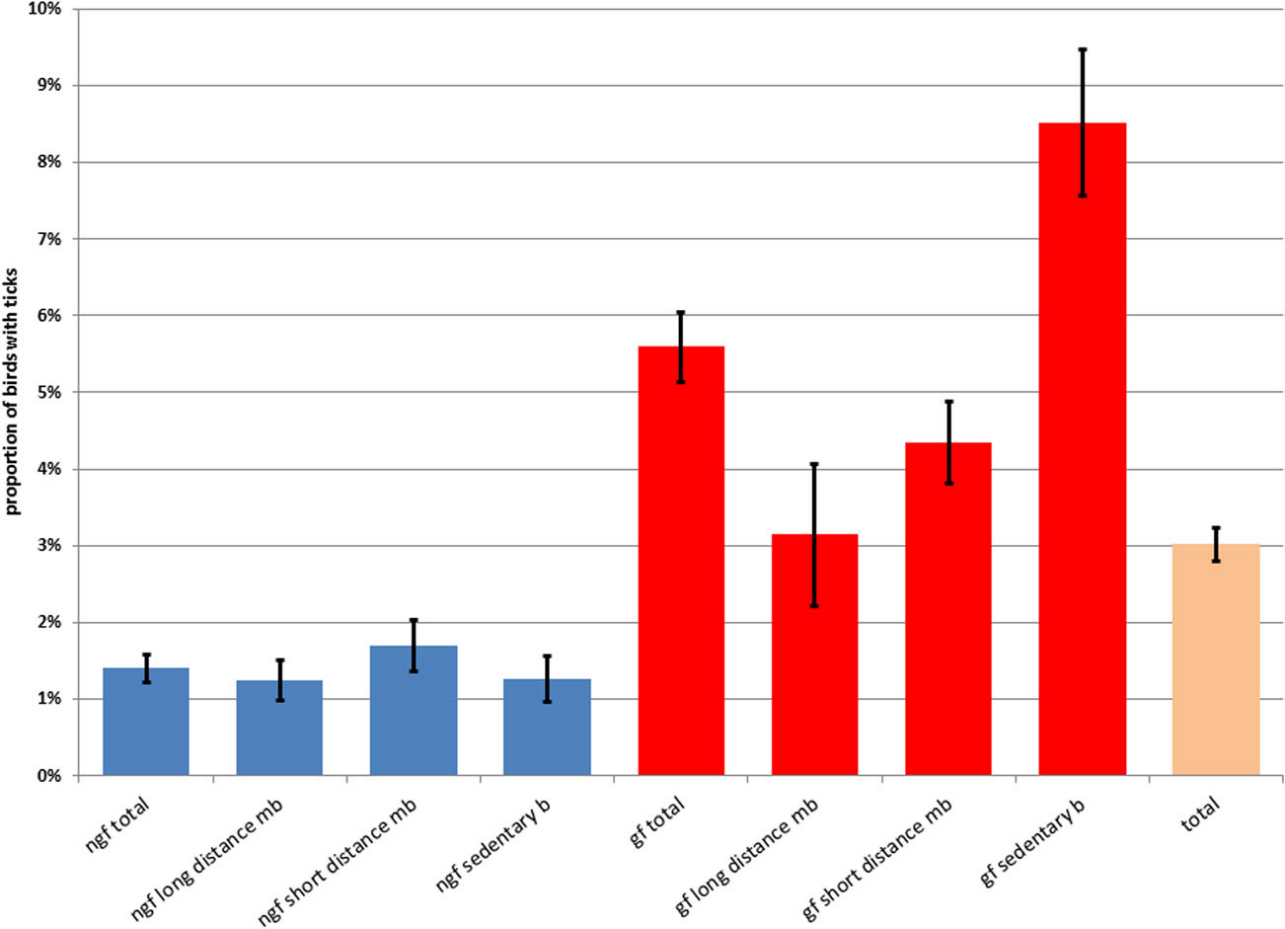
Proportion of birds with ticks related to migration and feeding type. *b* birds, *mb* migratory birds, *gf* ground feeders, *ngf* non-ground feeders

In total, 892 birds infested with ticks were found. After removing all datasets with missing data, especially without the total number of captured birds, 725 tick-infested birds, captured during ringing of 23,949 birds, could be further analysed for infestation rates, and a mean rate of 3.01 % (CI 2.80–3.24) was found. Most of the ticks were collected at the head around beak, eye and ear. The infestation rates were 5.59 % (CI 5.13–6.08) in ground feeders and 1.40 % (CI 1.22–1.61) in non-ground feeders. There is a significant difference between both groups (OR 4.16, *p* < 0.01, Fig. 1; Table 2). Referring to migration behaviour, the infestation rates were 4.29 % (CI 3.84–4.78 %) in sedentary birds and 2.45 % (CI 2.22–2.70) in migratory birds (OR 1.78, *p* < 0.01). Short-distance migratory birds were affected with higher rates (3.03 %, CI 2.71–3.38) than long-distance migratory birds (1.55 %, CI 1.27–1.88) as illustrated in Fig. 1 (OR 1.98, *p* < 0.01) in the groups of ground feeders and non-ground feeders, and Table 2.

**Table 2:** Result of the pairwise comparison for feeding and migration type

Comparison between groups	OR	Lower.ci	Upper.ci	Fisher test two-sided *P* value
Sedentary birds to migratory birds	1.78	1.53	2.08	<0.01
Sedentary birds to short-distance migratory birds	1.43	1.22	1.69	<0.01
Sedentary birds to long-distance migratory birds	2.84	2.26	3.60	<0.01
Short-distance migratory birds to others	1.01	0.87	1.18	0.91
Short-distance migratory birds to long-distance migratory birds	1.98	1.57	2.51	<0.01
Long-distance migratory birds to others	0.43	0.34	0.53	<0.01
Ground feeders to non-ground feeders	4.16	3.52	4.92	<0.01
Sedentary birds: ground feeders to non-ground feeders	7.25	5.36	9.95	<0.01
Short-distance migratory birds: ground feeders to non-ground feeders	2.64	2.04	3.44	<0.01
Long-distance migratory birds: ground feeders to non-ground feeders	2.57	1.63	3.98	<0.01

**Fig. 2:**
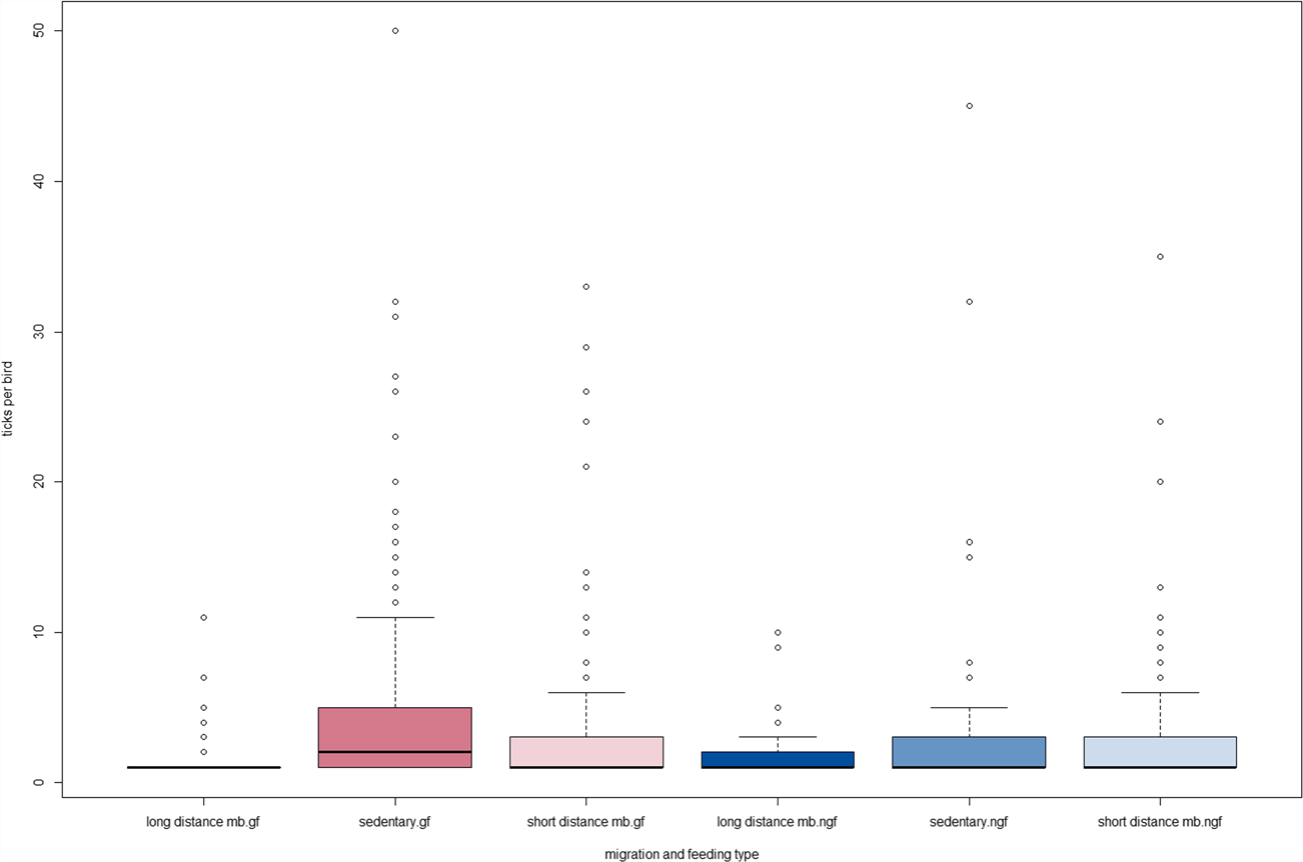
Ticks per bird related to migration and feeding type. *mb* migratory birds, *gf* ground feeders, *ngf* non-ground feeders

In the next steps, all 892 birds which were infested by ticks were included. In order to analyse tick infestation referring to data from birds, complete data sets were available from 838 tick-infested birds and were analysed with the following results: The mean tick infestation per bird was 4.2 ticks in the ground feeder group and 3.0 in the non-ground feeder group (Table 3). Referring to migration behaviour, remarkable differences were seen: Sedentary birds had an average of 4.7 ticks per bird, short-distance migratory birds 3.2 ticks and long-distance migratory birds only 2.0 ticks per bird (Table 4). Figure 2 highlights that in every group of birds referring to migration or feeding behaviour, some single individuals were infested by 10 up to 55 ticks per bird.

**Table 3:** Ticks collected from birds—non-ground feeders compared to ground feeders

Feeding	Tick growth stage	Birds	Ticks	Ticks per bird	Tick species					
					*Ixodes* spp.^a^	*I. ricinus*	*I. canisuga*	*I. hexagonus*	*I. lividus*	*Haemaphy-salis* spp.
ngf	Larvae	76	269	3.5	172	97	0	0	0	0
ngf	Nymphs	179	510	2.8	1	503	2	2	0	2
ngf	Female	12	28	2.3	0	12	1	0	15	0
Summary ngf		267	807	3.0	173	612	3	2	15	2
gf	Larvae	252	784	3.1	499	285	0	0	0	0
gf	Nymphs	309	1570	5.1	0	1569	0	0	0	1
gf	Female	8	32	4.0	0	25	7	0	0	0
gf	Male	2	2	1.0	0	2	0	0	0	0
Summary gf		571	2388	4.2	499	1881	7	0	0	1
Total		838	3195	3.8	672	2493	10	2	15	3

*ngf* non-ground feeders, *gf* ground feeders

^a^Further differentiation was not possible

**Table 4:** Ticks collected from birds—compared to migration type

Migration type	Tick growth stage	Birds	Ticks	Ticks per bird	Tick species					
					*Ixodes* spp.^a^	*I. ricinus*	*I. canisuga*	*I. hexagonus*	*I. lividus*	*Haemaphy-salis* spp.
Long-distance	Larvae	31	79	2.5	56	23	0	0	0	0
Long-distance	Nymphs	99	175	1.8	0	173	0	1	0	1
Long-distance	Female	6	18	3.0	0	3	0	0	15	0
Long-distance summary		136	272	2.0	56	199	0	1	15	1
Short-distance	Larvae	93	257	2.8	120	137	0	0	0	0
Short-distance	Nymphs	154	546	3.5	1	543	0	0	0	2
Short-distance	Female	7	19	2.7	0	12	7	0	0	0
Short-distance summary		254	822	3.2	121	692	7	0	0	2
Sedentary	Larvae	204	717	3.5	495	222	0	0	0	0
Sedentary	Nymphs	235	1359	5.8	0	1356	2	1	0	0
Sedentary	Female	7	23	3.3	0	22	1	0	0	0
Sedentary	Male	2	2	1.0	0	2	0	0	0	0
Sedentary summary		448	2101	4.7	495	1602	3	1	0	0
Total		838	3195	3.8	672	2493	10	2	15	3

^a^Further differentiation was not possible

In the next analysis, all 892 birds were included. Table 5 shows the bird species with 10 or more individuals infested by ticks. Two hundred forty-five birds were blackbirds (*Turdus merula*), followed by 99 song thrushes (*Turdus philomelos*), 91 robins (*Erithacus rubecula*) and 71 blackcaps (*Sylvia atricapilla*). Altogether the 732 birds represent 14 species with 10 or more tick-infested individuals, and the remaining 160 tick-infested birds represent the other 34 species with tick-infested individuals only in single cases.

**Table 5:** Most infested bird species (10 or more individuals infested by ticks)

Species		No. tick infested birds	No. ticks	Migrating behaviour	Feeding behaviour
Blackbird	*Turdus merula*	245	1383	Sedentary bird	Ground feeder
Song thrush	*Turdus philomelos*	99	328	Short-distance migratory bird	Ground feeder
Robin	*Erithacus rubecula*	91	330	Sedentary bird	Ground feeder
Black cap	*Sylvia atricapilla*	71	165	Short-distance migratory bird	Non-ground feeder
Great tit	*Parus major*	41	208	Sedentary bird	Non-ground feeder
Reed warbler	*Acrocephalus scirpaceus*	35	54	Long-distance migratory bird	Non-ground feeder
Marsh warbler	*Acrocephalus palustris*	31	56	Long-distance migratory bird	Ground feeder
Dunnock	*Prunella modularis*	29	187	Short-distance migratory bird	Non-ground feeder
Bullfinch	*Pyrrhula pyrrhula*	22	69	Sedentary bird	Ground feeder
Whitethroat	*Sylvia communis*	17	25	Long-distance migratory bird	Non-ground feeder
Chiffchaff	*Phylloscopus collybita*	15	17	Short-distance migratory bird	Ground feeder
Chaffinch	*Fringilla coeleps*	14	60	Short-distance migratory bird	Ground feeder
Wren	*Troglodytes troglodytes*	12	35	Sedentary bird	Ground feeder
Tree pipit	*Anthus trivialis*	10	28	Long-distance migratory bird	Ground feeder
Total		732	2945		

The highest proportions of tick-infested birds were again found in blackbirds (*Turdus merula*), and song thrushes (*Turdus philomelos*), followed by bullfinches (*Pyrrhula pyrrhula*). The lowest proportions were found in siskin (*Carduelus spinus*), tree sparrows (*Passer montanus*) and reed buntings (*Emberiza schoeniclus*).

In the years 2009 and 2010, ticks were collected year-round, in 2008 only from July to December. Therefore, only the years 2009 and 2010 could be compared in this study. A remarkable difference in the number of ticks per bird was only seen in the group of short-distance migratory birds, which were non-ground feeders: in 2009, 3.8 ticks were collected, whereas in 2010 only 1.9 ticks per bird could be collected (data not shown in detail). All other data did not differ remarkably between the years. For this reason, the data were interpreted in the discussion-section over the whole period of tick collection and not separated into 2009 and 2010.

The age of the birds was only recorded for tick-infested birds. These birds were divided into two groups: juvenile birds (up to one year) and adult birds. Four hundred forty-three juvenile birds and 422 adult birds with ticks were caught. No statistical differences between the two groups were noticed. For 27 birds, it was not possible to determine the age (data not shown in detail).

### Ticks

From 2008 to 2010, 3195 ticks were collected, identified and connected to the bird data (Tables 3 and 4). The most frequently detected tick species was *Ixodes* (*I*.) *ricinus* (78.02 %). 21.03 % of the ticks (especially engorged larvae) could only be identified as *Ixodes* spp. Among the 2520 ticks identified as different *Ixodes* spp., only 1.07 % belonged to the species *I. hexagonus*, *I. canisuga* or *I. lividus*. Only three nymphs from three different birds were identified as *Haemaphysalis* spp., other species like *Hyalomma* spp. or *Dermacentor* spp. were not found.

Birds were mostly infested by nymphs (65.10 %), followed by larvae (32.96 %). Only 1.94 % of the bird-infesting ticks were female or male adult ticks.

Only 12 of 838 tick-infested birds were infested with other than *I. ricinus* or not further differentiated *Ixodes* spp. (mostly engorged larvae). Among these 12 birds, only three were infested with *Haemaphysalis* spp. (three birds, three ticks), the others with *I. canisuga* (four birds, 10 ticks), *I. hexagonus* (two birds, two ticks) and *I. lividus* (three birds, 15 ticks).

### Detection of pathogens

For pathogen screening, a total of 425 pools created from five homogenized tick samples (nymphs, female and male adult ticks, larvae were not included) were investigated. In case of positive results, single ticks of the positive pool were investigated.

In a first PCR-screening, all pools were investigated for TBEV-RNA as the most likely viral pathogen in ticks in Germany. The PCR was combined with an *Ixodes*-specific extraction control in a duplex assay. Successful nucleic acid extraction could be confirmed in all 425 pools. However, no TBEV-RNA could be co-amplified from those samples. Identical negative results were obtained using the broad-range PCR-assays for Flaviviruses, Simbuviruses and Lyssaviruses as well as the Sindbisvirus RT-qPCR. However, the positive control reactions confirmed that the assay and test conditions were suitable.

In contrast to the viral pathogens, the Pan-Borrelia real-time PCR determined 57 pools as *Borrelia* spp.-positive. Forty of the 57 *Borrelia* spp.-positive tick pools consisted of tick samples collected from two or more birds. From these 40 pools, all single ticks were analysed together with 45 larvae that had not been tested in pools before. Ninety-seven *Borrelia* spp.-positive nymphs and 27 *Borrelia* spp.-positive larvae were detected.

Seventeen *Borrelia* spp.-positive pools were not checked as single ticks because each of these pools consisted of ticks collected from one bird. The result of *Borrelia* spp.-positive ticks in these pools can theoretically match between 17 (only one tick in the pool was *Borrelia* spp.-positive) and a maximum of 85 (all five ticks in the pool were *Borrelia* spp. positive) and was not further differentiated. Together with the 97 *Borrelia* spp.-positive nymphs and 27 *Borrelia* spp.-positive larvae, investigated as single ticks, in total between 141 and 209 *Borrelia* spp.-positive ticks were found. In relation to the whole number of *Borrelia*-examined ticks (2125 nymphs and adults plus 45 larvae) 6.50–9.63 % of the ticks were found to be *Borrelia* spp.-positive.

*Borrelia* spp.-positive ticks were collected from 76 birds, representing 8.52 % of all tick-infested birds. Among the 76 birds, a majority of 54 individuals were blackbirds (*Turdus merula*), followed by 12 song thrushes (*Turdus philomelos*), and 10 individuals belonging to six species: pied flycatcher (*Ficedula hypoleuca*), bullfinch (*Pyrrhula pyrrhula*), dunnock (*Prunella modularis*), great tit (*Parus major*), long-tailed tit (*Aegithalos caudatus*) and marsh warbler (*Acrocephalus palustris*).

**Fig. 3:**
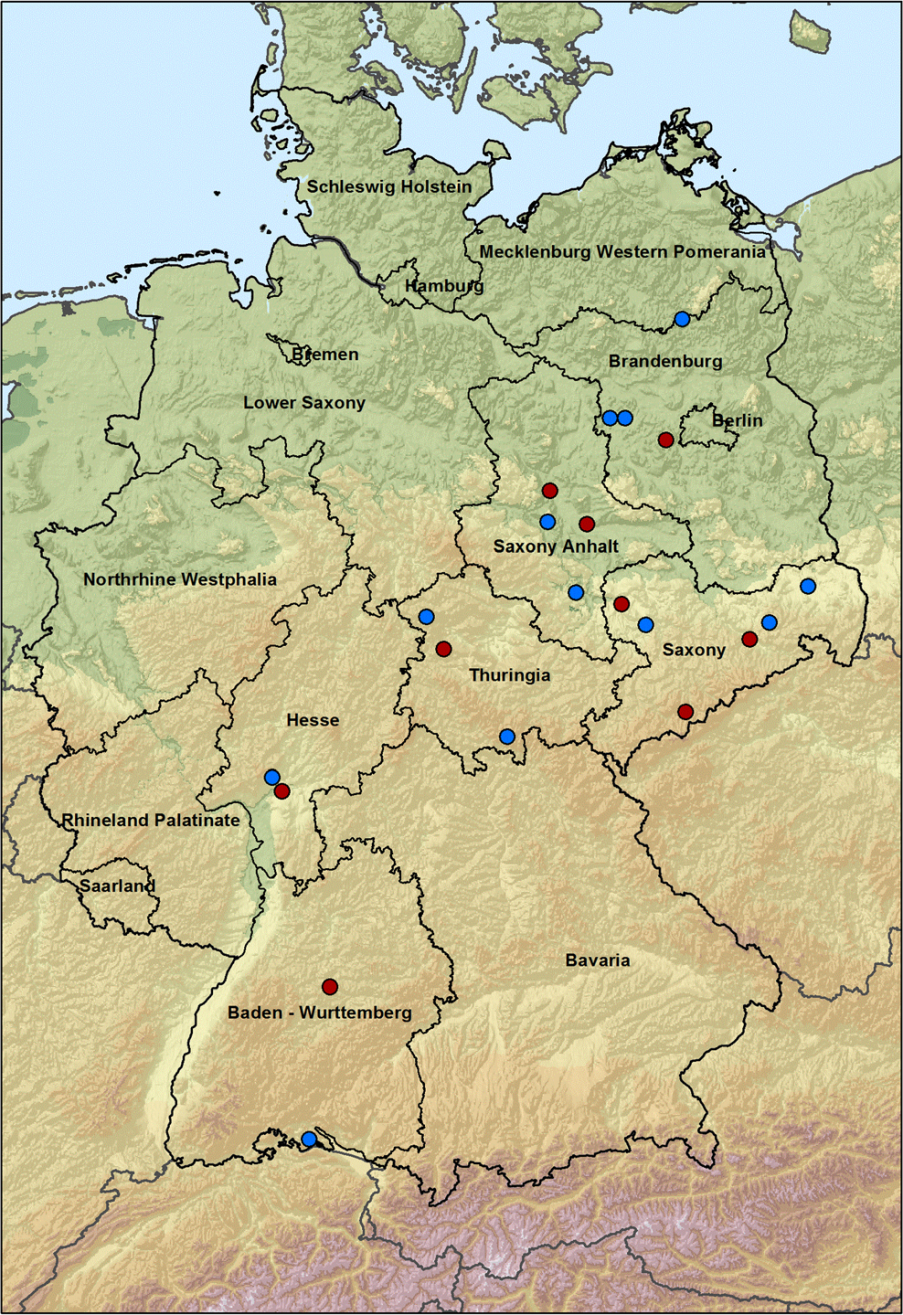
Bird and tick collecting places. *red*: *Borrelia* spp.-positive ticks were found, *blue*: no *Borrelia* spp.-positive ticks were found

Figure 3 shows the bird collecting places. All places where birds with *Borrelia* spp.-positive ticks were caught were marked with a red dot, places with *Borrelia* spp.-negative ticks were marked with a blue dot. Most birds with *Borrelia* spp.-positive ticks were caught in Mülverstedt, Thuringia (18 birds, 11.8 % of all tick-wearing birds caught at this place), Dresden, Saxony (14 birds, 13.5 % of all tick-bearing birds caught at this place), Frankfurt, Hesse (11 birds, 12.8 % of all tick-wearing birds caught at this place), and Annaberg-Buchholz, Saxony (10 birds, 13.7 % of all tick-bearing birds caught at this place).

Three places with *Borrelia* spp.-positive ticks (Mülverstedt, Thuringia, Annaberg-Buchholz and Dresden, both Saxony) were chosen because ticks from birds were collected over 2 years at these places. Here, a species differentiation of *Borrelia* spp. was performed. Forty-eight single ticks were tested, and, in spite of the very limited amount of original material, *Borrelia* strains from 18 ticks were successfully determined (Table 6). In total, the most common species at these three places was *B. garinii* (10 samples, 5 different strains), followed by *B. valaisiana* (6 samples, 2 different strains) and one strain each of *B. burgdorferi* s.s. and *B. afzelii*.

**Table 6:** *Borrelia*-species differentiation, detected in ticks collected at three selected places

Place	Year	Borrelia species	Strains	Annotation
Mülverstedt	2009	1× *B. afzelii*		
Mülverstedt	2009	2× *B. garinii*	2 strains	
Mülverstedt	2009	1× *B. burgdorferi* s.s.		
Mülverstedt	2009	1× no result		
Mülverstedt	2010	1× *B. garinii*		The same stain as in 2009
Mülverstedt	2010	1× B. valaisiana		The same strain also in Dresden
Mülverstedt	2010	9× no result		
Annaberg-Buchholz	2008	3× *B. valaisiana*	2 strains	
Annaberg-Buchholz	2008	1× *B. garinii*		
Annaberg-Buchholz	2008	3× no result		
Annaberg-Buchholz	2010	3× *B. garinii*	3 strains	different from 2008
Annaberg-Buchholz	2010	1× *B. valaisiana*		The same strain as in 2008
Annaberg-Buchholz	2010	5× no result		
Dresden	2009	2× *B. garinii*		
Dresden	2009	5× no result		
Dresden	2010	1× *B. garinii*		The same strain as in 2009
Dresden	2010	1× *B. valaisiana*		The same strain also in Mülverstedt
Dresden	2010	7× no result		

## Discussion

For many years, it has been well known that birds can serve as hosts for ticks, and depending on their specific biology they can cover smaller or greater distances, for example a long-distance migratory bird up to 1600 km non-stop across the Sahara (Hoogstraal and Kaiser 1961; Hoogstraal et al. 1961, 1963). Ticks and tick-borne pathogens can thus be transported to new non-endemic areas. In Sweden, 13 tick species have been listed, at least some of them occasionally, and the potential introduction of exotic pathogens is considered to be evident. It is mainly caused by the large number of people, animals and materials transported across the world, but also by migratory birds (Jaenson et al. 1994).

In most of the existing studies, only a small number of migratory birds was found to be infested with ticks. In Sweden 8 % (Elfving et al. 2010), in Great Britain 8 % (Jameson et al. 2012), in Greece (Antikythira) and Italy (Capri) 5 % (Lindeborg et al. 2012), in Finland 2.1 % of the birds were infested, on average with two ticks per bird (Nuorteva and Hoogstraal 1963). Our results with 3 % tick-infested birds (2.45 % of the migratory birds, 4.29 % of the sedentary birds) were in the same range. For Finland, extrapolated on the whole number of 600 million migratory birds, this means that every year nearly 25 millions of ticks are imported by birds (Nuorteva and Hoogstraal 1963). In the USA, more than 3000 birds were checked for ticks around the Gulf of Mexico. 3.56 % were infested with ticks (seven *Amblyomma* spp., one *Ixodes* spp.), and extrapolation of these findings suggests that four to 39 million exotic neotropical ticks could be transported to the USA annually by migratory songbirds with uncertain consequences for humans and animals (Cohen et al. 2015). For Europe such hypothesis should be handled with care: Most of the ticks found on birds were *Ixodes* spp.. Larvae need 2–5 days and nymphs 2–7 days for the blood meal, which limits the transportation distance. Birds need five and more days from Southern France to Iceland; however, the time span is strongly influenced by the weather conditions (Mehl et al. 1984). The average daily distance of small migratory bird species included in this study is about 60 to 75 km with a maximum of 150 km (Alerstam and Lindström 1990; Ellegren 1993; Berthold 2012). Depending on the bird species at least 6 to 14 days are needed to migrate from Northern African countries to Germany. In addition, the birds often stay at resting grounds for several days which further increases the migration time.

In contrast to *Ixodes* spp. *Hyalomma marginatum* needs up to 12–27 days for the blood meal (Balashov 1972), and in this time span it is possible that ticks can be transported from Africa to Europe. This was e.g., confirmed by the detection of *Hyalomma marginatum* in Norway (Mehl et al. 1984), Finland (Nuorteva and Hoogstraal 1963) and Sweden (Brinck et al. 1965) as well as in Great Britain (Jameson et al. 2012), Italy and Greece (Molin et al. 2011; Lindeborg et al. 2012; Wallmenius et al. 2014). In our investigations, no *Hyalomma marginatum* ticks were found. Thus, it can be concluded that *Ixodes* spp. collected from long-distance migratory birds were most likely acquired at a stopover place or near the place birds were caught for tick collection. Thus, a pathogen transfer by these ticks for example from Africa is not very likely.

Ground-feeder birds, migratory as well as sedentary birds, spend a predominant part of their live span in potential tick habitats and have a higher chance to be infested by ticks than birds that do not feed on the ground (Brinck et al. 1965; Cohen et al. 2015). This is in accordance with our results that demonstrated significant differences of tick infestation between ground feeders and non-ground feeders (5.59 % versus 1.40 % infested birds). Especially passerine birds, and in Europe the blackbird (*Turdus merula*) appear to be very important for harbouring ticks (Špitalská et al. 2006; Hasle 2013; Sándor et al. 2014). For Germany, our study confirmed that the blackbird (*Turdus merula*) plays a very important role. However, of the 14 most strongly infested bird species, nine species were migratory birds and able to transport ticks and pathogens to new potential foci, for example from the Mediterranean Sea to Germany. The importance of migratory birds for tick transportation could also be shown in the Danube delta by Sándor et al. (2014) where 92.25 % of the collected ticks were *I. ricinus*. This was also the predominant species in our investigations. Among the 3195 ticks, only three *Haemaphysalis* spp. ticks were detected, all the others belonged to the *Ixodes* spp., mostly *I. ricinus*. In our study in most cases, birds were infested with a low number of ticks per bird, i.e., between one and five. A mean infestation rate of two ticks per bird was found in winter in the Czech Republic (Literak et al. 2007), and six ticks per bird from spring to autumn in the Czech Republic and Slovakia (Taragel’ová et al. 2005). An influence on bird condition can thus be excluded. Norte et al. (2013) observed a significantly reduced general condition and weight loss in blackbirds (*Turdus merula*) infested with up to 10 or more larvae. In our study, the highest infestation in one bird was 55 ticks; however, such a high infestation rate is very seldom (Brinck et al. 1965).

Ticks on birds can be infected with a varying number of tick-borne pathogens, like *Borrelia* spp. (Anderson et al. 1986; Olsén et al. 1995; Gylfe et al. 2000; Comstedt et al. 2006; Poupon et al. 2006; Franke et al. 2010a, b), *Babesia* spp. (Hildebrandt et al. 2011), *Rickettsia* spp. (Elfving et al. 2010), *Anaplasma* and *Ehrlichia* spp. (Bjöersdorff et al. 2001; Hildebrandt et al. 2011). Only little is known about viral pathogens found in ticks from birds. Waldenström et al. (2007) checked 13,260 migratory birds in Sweden, 447 (3.4 %) birds were infested with ticks but only four birds were infested with six TBEV-infected ticks. It was concluded that TBEV can be distributed by tick-infested migratory birds in spite of the very low number of TBEV positive ticks detected. These findings were confirmed in Estonia (Geller et al. 2013), Switzerland (Lommano et al. 2014) and Latvia (Kazarina et al. 2015). Ernek et al. (1968) detected TBEV antibodies in sera from blackbirds (*Turdus merula*) and sparrows (*Passer domesticus*) as well as TBEV in ticks from blackbirds (*Turdus merula*). Other bird species might have a high natural resistance against TBEV. Experimental infections of buzzards (*Buteo buteo*), kestrels (*Falco tinnunculus*) (Rehaček et al. 1963), great tits (*Parus major*) (Grešíková et al. 1962) or pheasants (*Phasianus colchicus*) (Nosek et al. 1962) induced only in some cases TBEV-specific antibodies; neither viraemia nor clinical signs were observed. In our investigations, no TBEV-RNA could be detected. However, the most infested species blackbird (*Turdus merula*) could serve as transportation host for TBEV-infected ticks according to Ernek et al. (1968).

Hubálek (2004) emphasized that some species of migratory birds play an important role for circulation and ecology of some arboviruses like Sindbis virus or some *Flaviviridae*. Tick-borne *Bunyaviridae* in wild birds were not amplified, no clinical signs occurred, but the virus was detected in the blood for several days, which might be sufficient for an effective transportation to a new area (Hubálek et al. 1982). It was predicted that natural foci of tick-borne *Bunyaviridae* in Europe might spread from the south up to Switzerland, Austria and Germany, provided that winters in the next years are mild and summers are dry (Hubálek 2009). Crimean-Congo haemorrhagic fever virus (CCHFV), mostly found in *Hyalomma* spp., could adapt to other vectors (Bente et al. 2013). It might be spread to the north via animal trade and migratory birds, viraemia in the birds is not a precondition (Bente et al. 2013). Lindeborg et al. (2012) collected ticks from migratory birds on Capri (Italy) and Antikythira (Greece), 5 % of the birds were infested with ticks, mostly *Hyalomma marginatum*, only one bird was infested with three CCHFV virus positive *Hyalomma marginatum*.

Estrada-Peña et al. (2012) discussed CCHFV distribution, the lack of knowledge about vector competence of other tick species than *Hyalomma marginatum*, and generally recommended more empirical studies on the importance of birds for distribution of infected ticks and survival chances of the pathogens in new and up to now non-endemic areas. It is possible that mosquito-borne pathogens can adapt to ticks as vectors. Hagman et al. (2014) checked 747 ticks from migratory birds for West Nile virus (WNV), but all ticks were negative. Hubálek and Rudolf (2012) listed 27 tick-borne viruses in Europe, some of them are associated with birds. TBEV was detected in some bird species, like the common redpoll (*Acanthis flammea*) and the sparrow (*Passer domesticus*); viraemia after experimental infection was observed in some bird species like the common quail (*Coturnix coturnix*) or the mallard (*Anas plathyrhynchos*). Birds were not susceptible for experimental infection with CCHFV. Experimental infection with Bunyavirus Bhanja was not lethal for passerine birds. Some bird species (European starling, *Sturnus vulgaris*; chaffinch, *Fringilla coelebs*) can serve as hosts for orbivirus Tribeć without clinical symptoms, the same applies to orthobunyavirus Bahig and passerine birds. In many cases, only little is known about virus and birds. For distribution of Thogotovirus migratory birds can also be important (Calisher et al. 1987; Hubálek and Rudolf 2012).

In our study, the screening results for viral pathogens (Flavivirus-RT-PCR, Simbuvirus-RT-qPCR, Lyssavirus-RT-qPCR, Sindbisvirus-RT-qPCR) in ticks from birds in Germany were all negative. It can be concluded that in our study, all checked birds could not be detected as competent hosts or transportation hosts for these viral pathogens. Nevertheless, screening of ticks and birds for viral pathogens should be continued, especially with broad-range PCR methods as an early warning system.

Four *Borrelia* spp. were detected in ticks from birds (Table 6), distributed at nine of the 25 tick collecting places (Fig. 3). Birds, especially blackbirds (*Turdus merula*) and song trushes (*Turdus philomelos*), play an important role in the distribution of *Borrelia* spp. (Humair et al. 1993; Olsén et al. 1995; Taragel’ová et al. 2008), serve as reservoir hosts (Lommano et al. 2014), and can establish new *Borrelia* foci. In our study also rodent-associated *B. afzelii* was found in ticks from birds and confirmed the findings of Olsén et al. (1995), Franke et al. (2010b) and Geller et al. (2013) that also rodent-associated *B. afzelii* strains can be harboured in ticks feeding on birds. Gylfe et al. (2000) found reactivation of *Borrelia* infections in migratory birds caused by stress factors, which could explain the distribution of *Borrelia* spp. over longer distances and the infection of a new so far non-infected tick population. Among the most strongly infested birds besides blackbirds (*Turdus merula*) as a sedentary bird species, nine species were registered as short- or long-distance migratory birds (Table 5) that have the potential to distribute *Borrelia* spp. as described in new previously non-infected areas under stress conditions (Gylfe et al. 2000). In conclusion, not only blackbirds (*Turdus merula*) and other passerine birds should be examined for epidemiological studies of *Borrelia* spp. but also migratory bird species with relevant tick infestation.

To our knowledge, this was the first study about ticks and birds including such a large number of birds and ticks at different places in six federal states in Germany. In our investigations of birds, all collected tick species were well known in Germany and no unexpected exotic species was detected. For the first time, ticks from birds in Germany were also investigated for a wide range of viral pathogens. This was possible due to the established broad-range PCR systems for different virus genera. Nevertheless, no positive results were seen in this study, but cannot be excluded for the future. In addition, a high relevance of birds for the epidemiology of *Borrelia* spp. could be confirmed. Blackbirds (*Turdus merula*) but also some species of migratory birds highly infested with ticks should be focused for further investigations of this topic.

It is further suggested that short-distance migratory birds should be checked frequently for ticks in the future. Their specific biology allows them to transport ticks with pathogens for example from around the Mediterranean Sea to Germany within a few days. In case of good weather conditions, progressing climate change and presence of susceptible hosts for ticks and pathogens new viral endemic foci can be generated within a short time.

Estrada-Peña et al. (2012) recommended more empirical studies on the importance of birds for distribution of infected ticks and survival chances of the pathogens in new and so far non-endemic areas. Hubálek and Rudolf (2012) suggested that an active search for new tick-borne viruses or for new pathogenic variants of the known tick-borne viruses in Europe should be continued because some of them may often pass unnoticed or be misdiagnosed. For the future, long-term studies in the field including pathogens, vectors, hosts, microclimate and habitat structure are needed and should be combined with laboratory investigations on the transmission competence of various hosts to improve the knowledge about viral tick-borne diseases (Estrada-Peña and de la Fuente 2014).

## References

[CR1] Alerstam T, Lindström A, Gwinner E (1990). Optimal bird migration: the relative importance of time, energy and safety. Bird migration: the physiology and ecophysiology.

[CR2] Anderson JF, Russell CJ, Magnarelli LA, Hyde FW (1986). Involvement of birds in the epidemiology of the Lyme disease agent *Borrelia burgdorferi*. Infect Immun.

[CR3] Balashov YS (1972). A translation of “Bloodsucking ticks (Ixodoidea)—vectors of diseases of man and animals”. Misc Publ Ent Soc Am.

[CR4] Becker N, Jöst H, Ziegler U, Eiden M, Höper D, Emmerich P, Fichet-Calvet E, Ehichioya DU, Czajka C, Gabriel M, Hoffmann B, Beer M, Tenner-Racz K, Racz P, Günther S, Wink M, Bosch S, Konrad A, Pfeffer M, Groschup MH, Schmidt-Chanasit J (2012). Epizootic emergence of Usutu virus in wild and captive birds in Germany. PLoS One.

[CR5] Bente DA, Forrester NL, Watts DM, McAuley AJ, Whitehouse CA, Bray M (2013). Crimean-Congo hemorrhagic fever: history, epidemiology, pathogenesis, clinical syndrome and genetic diversity. Antivir Res.

[CR6] Berthold P (2012). Vogelzug—eine aktuelle gesamtübersicht.

[CR7] Bjöersdorff A, Bergström S, Massung RF, Haemig PD, Olsén B (2001). *Ehrlichia*-infected ticks on migrating birds. Emerg Infect Dis.

[CR8] Brinck P, Svedmyr A, von Zeipel G (1965). Migrating birds at Ottenby Sweden as carriers of ticks and possible transmitters of tick-borne encephalitis virus. OIKOS.

[CR9] Calisher CH, Karabatsos N, Filipe AR (1987). Antigenic uniformity of topotype strains of Thogoto virus from Africa, Europe and Asia. Am J Trop Med Hyg.

[CR10] Cohen EB, Auckland LD, Marra PP, Hamer SA (2015). Avian migrants facilitate invasions of Neotropical ticks and tick-borne pathogens into the United States. Appl Environ Microbiol.

[CR11] Comstedt P, Bergström S, Olsén B, Garpmo U, Marjavaara L, Mejlon H, Barbour AG, Bunikis J (2006). Migratory passerine birds as reservoirs of Lyme Borreliosis in Europe. Emerg Infect Dis.

[CR12] Eckert J, Friedhoff KT, Zahner H, Deplazes P (2008) Lehrbuch der Parasitologie für die Tiermedizin, 2nd edn. Enke Verlag in MVS Medizinverlage, Stuttgart. 375-392, 540

[CR13] Elfving K, Olsen B, Bergström S, Waldenström J, Lundkvist A, Sjöstedt A, Mejlon H, Nilsson K (2010). Dissemination of spotted fever Rickettsia agents in Europe by migrating birds. PLoS ONE.

[CR14] Ellegren H (1993). Speed of migration and migratory flight length of passerine birds ringed during autumn migration in Sweden. Ornis Scand.

[CR15] Ernek E, Kožuch O, Lichard M, Nosek J (1968). The role of birds in the circulation of tick-borne encephalitis virus in the Tribeč region. Acta Virol.

[CR16] Estrada-Peña A (2009). Tick-borne pathogens, transmission rates and climate change. Front Biosci.

[CR17] Estrada-Peña A, Bouattour A, Camicas JL, Walker AR (2004). Ticks of domestic animals in the mediterranean region.

[CR18] Estrada-Peña A, Jameson L, Medlock J, Vatansever Z, Tishkova F (2012). Unraveling the ecological complexities of tick-associated crimean-congo hemorrhagic fever virus transmission: a gap analysis for the western palearctic. Vector Borne Zoonotic Dis.

[CR19] Estrada-Peña A, de la Fuente J (2014). The ecology of ticks and epidemiology of tick-borne viral diseases. Antivir Res.

[CR20] Fischer M, Schirrmeier H, Wernike K, Wegelt A, Beer M, Hoffmann B (2013). Development of a pan-Simbu real-time reverse transcriptase PCR for the detection of Simbu serogroup viruses and comparison with SBV diagnostic PCR systems. Virol J.

[CR21] Fischer M, Freuling CM, Müller T, Wegelt A, Kooi EA, Rasmussen TB, Voller K, Marston DA, Fooks AR, Beer M, Hoffmann B (2014). Molecular double-check strategy for the identification and characterization of European Lyssaviruses. J Virol Methods.

[CR22] Franke J, Fritzsch J, Tomaso H, Straube E, Dorn W, Hildebrandt A (2010). Coexistence of pathogens in host-seeking and feeding ticks within a single natural habitat in central Germany. Appl Environ Microbiol.

[CR23] Franke J, Meier F, Moldenhauer A, Straube E, Dorn W, Hildebrandt A (2010). Established and emerging pathogens in *Ixodes ricinus* ticks collected from birds on a conservation island in the Baltic Sea. Med Vet Entomol.

[CR24] Geller J, Nazarova L, Katargina O, Leivits A, Järvekülg L, Golovljova I (2013). Tick-borne pathogens in ticks feeding on migratory passerines in western part of Estonia. Vector Borne Zoonotic Dis.

[CR25] Grešíková M, Nosek J, Rehaček J, Albrecht P (1962). The role of birds in a natural focus of tick-borne encephalitis II. Experimental infection of Great Tits (Parus major L.) with Tick-borne Encephalitis Virus. J Hyg Epidemiol Microbiol Immunol.

[CR26] Gylfe Å, Bergström S, Lundström J, Olsén B (2000). Reactivation of Borrelia infection in birds. Nature.

[CR27] Hagman K, Barboutis C, Ehrenborg C, Fransson T, Jaenson TGT, Lindgren PE, Lundkvist Å, Nyström F, Waldenström J, Salaneck E (2014). On the potential roles of ticks and migrating birds in the ecology of West Nile virus. Infect Biol Epidemiol.

[CR28] Hasle G (2013). Transport of ixodid ticks and tick-borne pathogens by migratory birds. Front Cell Infect Microbiol.

[CR29] Hildebrandt A, Franke J, Schmoock G, Pauliks K, Krämer A, Straube E (2011). Diversity and coexistence of tick-borne pathogens in Central Germany. J Med Entomol.

[CR30] Hoffmann B, Scheuch M, Höper D, Jungblut R, Holsteg M, Schirrmeier H, Eschbaumer M, Goller KV, Wernike K, Fischer M, Breithaupt A, Mettenleiter TC, Beer M (2012). Novel orthobunyavirus in Cattle, Europe, 2011. Emerg Infect Dis.

[CR31] Hoogstraal H, Kaiser MN (1961). Ticks from european-asiatic birds migrating through egyppt into africa. Science.

[CR32] Hoogstraal H, Kaiser MN, Traylor MA, Gaber S, Guindy E (1961). Ticks (*Ixodoidea*) on birds migrating from Africa to Europe and Asia. Bull World Health Organ.

[CR33] Hoogstraal H, Kaiser MN, Traylor MA, Guindy E, Gaber S (1963). Ticks (*Ixodidae*) on birds migrating from europe and asia to africa, 1959-61. Bull World Health Organ.

[CR34] Hubálek Z (2004). An annotated checklist of pathogenic microorganisms associated with migratory birds. J Wildl Dis.

[CR35] Hubálek Z (2009). Biogeography of tick-borne Bhanja virus (bunyaviridae) in europe. Interdiscip Perspect Infect Dis.

[CR36] Hubálek Z, Černý V, Rödl P (1982). Possible role of birds and ticks in the dissemination of Bhanja virus. Folia Parasitol (Praha).

[CR37] Hubálek Z, Rudolf I (2012). Tick-borne viruses in Europe. Parasitol Res.

[CR38] Humair PF, Turrian N, Aeschlimann A, Gern L (1993). *Ixodes ricinus* immatures on birds in a focus of Lyme borreliosis. Folia Parasitol (Praha).

[CR39] Jaenson TGT, Tälleklint L, Lundqvist L, Olsen B, Chirico J, Mejlon H (1994). Geographical distribution, host associations, and vector roles of ticks (Acari: Ixodidae, Argasidae) in Sweden. J Med Entomol.

[CR40] Jameson LJ, Morgan PJ, Medlock JM, Watola G, Vaux AGC (2012). Importation of *Hyalomma marginatum*, vector of Crimean-Congo haemorrhagic fever virus, into the United Kingdom by migratory birds. Ticks Tick Borne Dis.

[CR41] Jöst H, Bialonski A, Storch V, Günther S, Becker N, Schmidt-Chanasit J (2010). Isolation and phylogenetic analysis of Sindbis viruses from mosquitoes in Germany. J Clin Microbiol.

[CR42] Johnson N, Wakeley PR, Mansfield KL, McCracken F, Haxton B, Phipps LP, Fooks AR (2010). Assessment of a novel real-time pan-flavivirus RT-polymerase chain reaction. Vector Borne Zoonotic Dis.

[CR43] Kazarina A, Japina K, Keišs O, Salmane I, Bandere D, Capligina V, Ranka R (2015). Detection of tick-borne encephalitis virus in I. Ricinus ticks collected from autumn migratory birds in Latvia. Ticks Tick Borne Dis.

[CR44] Klaus C, Hoffmann B, Hering U, Mielke B, Sachse K, Beer M, Süss J (2010). Tick-borne encephalitis (TBE) virus prevalence and virus genome characterization in field-collected ticks (*Ixodes ricinus*) in risk, non-risk, and former risk areas of TBE, and in ticks removed from humans in Germany. Clin Microbiol Infect.

[CR45] Lindeborg M, Barboutis C, Ehrenborg C, Fransson T, Jaenson TGT, Lindgren PE, Lundkvist Å, Nyström F, Salaneck E, Waldenström J, Olsen B (2012). Migratory birds, ticks, and Crimean-Congo hemorrhagic fever virus. Emerg Infect Dis.

[CR46] Literak I, Kocianova E, Dusbabek F, Martinu J, Podzemny P, Sychra O (2007). Winter infestation of wild birds by ticks and chiggers (Acari: Ixodidae, Trombiculidae) in the Czech Republic. Parasitol Res.

[CR47] Lommano E, Dvořák C, Vallotton L, Jenni L, Gern L (2014). Tick-borne pathogens in ticks collected from breeding and migratory birds in Switzerland. Ticks Tick Borne Dis.

[CR48] Mehl R, Michaelsen J, Lid G (1984). Ticks (Acari, Ixodides) on migratory birds in Norway. Fauna Norv Ser B.

[CR49] Molin Y, Lindeborg M, Nyström F, Madder M, Hjelm E, Olsen B, Jaenson TGT, Ehrenborg C (2011). Migratory birds, ticks, and Bartonella. Infect Ecol Epidemiol.

[CR50] Norte AC, Lobato DNC, Braga EM, Antonini Y, Lacorte G, Gonçalves M, Lopes de Carvalho I, Gern L, Núncio MS, Ramos JA (2013). Do ticks and Borrelia burgdorferi s.l. constitute a burden to birds?. Parasitol Res.

[CR51] Nosek J, Grešíková M, Rehaček J, Kožuch O, Albrecht P (1962). The role of birds in a natural focus of tick-borne encephalitis IV. Experimental infection of pheasants (Phasianus colchicus) with Tick-borne Encephalitis Virus. J Hyg Epidemiol Microbiol Immunol.

[CR52] Nuorteva P, Hoogstraal H (1963). The incidence of ticks (*Ixodoidea*, *Ixodidae*) on migratory birds arriving in finland during the spring of 1962. Ann Med Exp Biol Fenn.

[CR53] Olsén B, Jaenson TGT, Bergström S (1995). Prevalence of *Borrelia burgdorferi* sensu lato-infected ticks on migrating birds. Appl Environ Microbiol.

[CR54] Pfeffer M, Dobler G (2010). Emergence of zoonotic arboviruses by animal trade and migration. Parasit Vectors.

[CR55] Pfister K (2006) Arthropodenbefall bei Wiederkäuern, Arthropodenbefall bei Hund und Katze. In: Schnieder T (ed) Veterinärmedizinische Parasitologie, 6th edn. Parey, Stuttgart. 235–244. 521–533

[CR56] Poupon MA, Lommano E, Humair PF, Douet V, Rais O, Schaad M, Jenni L, Gern L (2006). Prevalence of *Borrelia burgdorferi* sensu lato in ticks collected from migratory birds in Switzerland. Appl Environ Microbiol.

[CR57] Rehaček J, Grešíková M, Nosek J, Albrecht P (1963). Experimental infection of the buzzard (Buteo buteo L.) and the kestrel (Falco tinnunculus L.) with tick-borne encephalitis virus. J Hyg Epidemiol Microbiol Immunol.

[CR58] Sándor AD, Mărcuţan DI, D’Amico G, Gherman CM, Dumitrache MO, Mihalca AD (2014). Do the ticks of birds at an important migratory hotspot reflect the seasonal dynamics of Ixodes ricinus at the migration initiation site? A case study in the Danube Delta. PLoS One.

[CR59] Schwaiger M, Cassinotti P (2003). Development of a quantitative real-time RT-PCR assay with internal control for the laboratory detection of tick borne encephalitis virus (TBEV) RNA. J Clin Virol.

[CR60] Špitalská E, Literák I, Sparagano AE, Golovchenko M, Kocianová E (2006). Ticks (Ixodidae) from passerine birds in the Carpathian region. Wien Klin Wochenschr.

[CR61] Strube C, Montenegro VM, Epe C, Eckelt E, Schnieder T (2010). Establishment of a minor groove binder-probe based quantitative real time PCR to detect *Borrelia burgdorferi* sensu lato and differentiation of *Borrelia spielmanii* by *osp*A-specific conventional PCR. Parasit Vectors.

[CR62] Taragel’ová V, Koči J, Hanincová K, Olekšák M, Labuda M (2005). Songbirds as hosts for ticks (Acari: Ixodidae) in Slovakia. Biologia.

[CR63] Taragel’ová V, Koči J, Hanincová K, Kurtenbach K, Derdáková M, Ogden NH, Literák I, Kocianová E, Labuda M (2008). Blackbirds and song thrushes constitute a key reservoir of Borrelia garinii, the causative agent of Borreliosis in central Europe. Appl Environ Microbiol.

[CR64] Waldenström J, Lundkvist A, Falk KI, Garpmo U, Bergström S, Lindegren G, Sjöstedt A, Mejlon H, Fransson T, Haemig PD, Olsen B (2007). Migrating birds and tickborne encephalitis virus. Emerg Infect Dis.

[CR65] Wallmenius K, Barboutis C, Fransson T, Jaenson TGT, Lindgren PE, Nyström F, Olsen B, Salaneck E, Nilsson K (2014). Spotted fever *Rickettsia* species in *Hyalomma* and *Ixodes* ticks infesting migratory birds in the European Mediterranean area. Parasit Vectors.

[CR66] Wilson AJ, Mellor PS (2009). Bluetongue in Europe: past, present and future. Philos Trans R Soc Lond B Biol Sci.

[CR67] World Health Organization (2004). The vector-borne human infections in Europe: their distribution and burden of public health.

[CR68] World Health Organization (2014). A global brief on vector-borne diseases.

